# Present and Future of AI-IoT-Based Healthcare Services for Senior Citizens in Local Communities: A Review of a South Korean Government Digital Healthcare Initiatives

**DOI:** 10.3390/healthcare12020281

**Published:** 2024-01-22

**Authors:** Dong-Jin Kim, Yun-Su Lee, Eun-Raye Jeon, Kwang Joon Kim

**Affiliations:** 1 Department of Digital Healthcare, Korea Health Promotion Institute, Seoul 04554, Republic of Korea; kemp75@khepi.or.kr (D.-J.K.); jv1000lys@khepi.or.kr (Y.-S.L.); 2 Department of Home Economics Education, Chonnam National University, Gwangju 61186, Republic of Korea; 3 College of Pharmacy, Chonnam National University, Gwangju 61186, Republic of Korea

**Keywords:** digital healthcare, senior citizens, artificial intelligence, internet of things

## Abstract

South Korea is promoting digital healthcare services in the public sector. One notable initiative is the “artificial intelligence and the internet of things (AI–IoT)-based healthcare project for senior citizens”, which was implemented by the Korea Health Promotion Institute (KHPI). This project utilized an IoT-based digital healthcare service that integrates information technology and screen-based AI speaker functions. Services through this project are intended for senior citizens aged 65 years (or older) who face challenges in visiting public healthcare institutions owing to limitations on outdoor activities, especially in the post-coronavirus 2019 era. This article shares the recent outcomes of this project and outlines the mid-to-long-term development strategies for this style of South Korean digital healthcare initiatives.

## 1. Introduction

In modern society, improvements in economic levels and changes in people’s lifestyle patterns attributed to environmental factors have led to the emergence of various metabolic disorders, such as hypertension and diabetes. Metabolic syndrome is a predictor of the risk of cardiovascular diseases and diabetes. Compared with healthy groups, the incidence of cardiovascular diseases is higher by approximately 60% in the group of people suffering from metabolic syndrome. Moreover, the risk of diabetes is higher by at least five times [1].

In line with the advancement of medical technology, new healthcare methods (exercise therapy, diet, etc.) have been developed in conjunction with a broad range of research for the treatment of diseases. And the cultural phenomenon of proactive wellbeing to promote “living healthier and longer” has led to an increased level of interest in the use of digital healthcare service in the management of the metabolic syndrome.

Digital healthcare is a collective term that encompasses all the services provided through the digitalization of processes associated with public health and medical services, such as disease prevention and treatment, medical care, and nursing. In the past, healthcare trends primarily emphasized diagnosis and treatment. However, today’s healthcare trends are shifting towards promoting wellbeing and prevention health [2]. With the accumulation of extensive data and advancements in AI, particularly through deep learning, digital healthcare services now possess the potential to predict diseases and complications while offering personalized services accessible at any time and place. This progress in digital healthcare is expected to significantly improve public health [3].

The world has undergone a new paradigm shift over the past 3 years, namely, familiarization with contactless living conditions and the acceleration of digital transformation, owing to the COVID-19 pandemic. In terms of healthcare, various opinions have been reported, and studies have been conducted on the convenience of healthcare services, such as contactless medical treatments and application (app)-based digital healthcare plans [4,5].

Meanwhile, in conjunction with infectious diseases, the COVID-19 pandemic and an aging society have emerged as new issues across the globe. As of 2021, the population of senior citizens aged 65 years (or older) in South Korea accounts for 16.5% of the national population. As this population proportion is anticipated to increase steadily to 20.3% by 2025, South Korea is expected to enter a super-aged society era [6]. This will lead to a rapid rise in the medical expenses of senior citizens, amplifying the socioeconomic burden. In 2020, individuals aged 65 and over accounted for 43.4% of the total medical expenses, with the medical costs per elderly person being approximately three times higher than those for the general population [7]. In other words, an increase in the percentage of the population of senior citizens aged ≥65 years means an increase in medical expenses and the occurrence of socioeconomic issues; hence the necessity for the prevention of chronic diseases.

In 2022, the new South Korean Government announced policy tasks which involve the expansion of an artificial intelligence (AI) and internet of things (IoT)-based healthcare project for senior citizens in the Fourth Industrial Revolution era. In addition, the Ministry of Health and Welfare (MOHW) (2023), based on its new-year work report, proposed a plan to expand the phases of the AI-IoT-based healthcare project for senior citizens. This is the first public-led contactless healthcare project to provide services by categorizing senior citizens into classes with different weakness levels as part of the plan for smart health support based on life cycle stages.

In line with this trend, this study aims to share the recent outcomes of the digital healthcare service for senior citizens which was implemented by the Korea Health Promotion Institute (KHPI) and outlines mid-to-long-term development strategies for this style of South Korean digital healthcare initiatives.

## 2. Results and Discussion

### 2.1. Digital Transformation and Public Health Centers in Local Communities in South Korea

Given the increasing level of interest in healthcare services using smartphones and applications, the number of wearable devices that can be linked to smartphones is increasing exponentially. Accordingly, the use of digital technology is recognized as innovation in healthcare services. Specifically, in South Korea, the smartphone distribution rate (as of 2016) was 91% and was the highest in the Asia–Pacific region. Moreover, as of 2021, the smartphone distribution rate among people aged 60 years and older was as high as 80%. In terms of public big data management for health and diseases and the latest information and communications technologies, South Korea has a competitive edge across the world [8,9,10]. Given the circumstances, digital transformation is also necessary for the health promotion and healthcare of residents. The COVID-19 pandemic enhanced citizens’ familiarity with public health centers in local communities. Public health centers implement various health promotion projects in addition to infectious disease control. Therefore, the health promotion system of public health centers also needs a paradigm shift, and restructuring healthcare services in local communities using digital technology is considered a natural course of action.

In fact, according to a recent publication, 911 adults who had completed a 6-month public mobile healthcare service project in 2016 produced positive results in terms of the physical activity continuation rate and health hazard factors (total cholesterol, systolic blood pressure, triglycerides, etc.) [11]. In addition, Park Na-yeong et al. verified the health improvement effect of the public mobile health (M-Health) project and reported a positive effect especially in the high-risk group [12]. Moreover, the service users tended to participate actively in healthcare instead of being passive and dependent on medical personnel, as in the past.

Meanwhile, the World Health Organization (WHO) emphasizes the need to guarantee sustainability and health equity, strengthen access to health information and services, and enforce digital healthcare strategies for innovation in disease prevention. Likewise, in the fifth National Health Plan (HP2030) of South Korea announced in 2021, the task of creating innovative information technology applications was added as a digital healthcare policy. This policy focuses on the establishment of an innovative technological system and access improvement [13]. The revised edition of HP2030 (2022) emphasizes the use of public digital healthcare services as a strategy to improve health equity and the strategy of using innovative information technology to expand service applications to digitally literate senior citizens. As mentioned in the introduction, the types of public medical services are changing rapidly owing to COVID-19, and senior citizens who have been neglected in the digital healthcare market due to their unfamiliarity with the use of digital technology are emerging as the main customer base [14].

Ultimately, a paradigm shift ought to be promoted for the health promotion of the services of public health centers in local communities based on the recognition of digital transformation as a current major trend.

### 2.2. Status of Domestic and International Digital Healthcare Services

Global healthcare companies are launching and operating services by analyzing and studying various areas of medical services based on their independent technological power. In the field of healthcare service, companies such as the wearable device (smart band) specialist Fit-bit^®^, and Noom^®^, a company providing customized coaching services on a healthcare platform, are offering individually customized services [14]. Regarding medical treatments, Insulia^®^, an insulin dosage calculation app for type 2 diabetes developed by the French company Voluntis^®^ (famous for digital therapeutics (DTx)), and Oleena^®^, a prescription mobile app for cancer patients, are available. In addition, Libre^®^, an app for noninvasive continuous glucose measurements and monitoring that has become available lately; Propeller^®^, an app for respiratory system management for (among others) asthma and chronic obstructive pulmonary disease, Somryst^®^; used for chronic insomnia and sleep control; and iRhythm^®^, an app that has marked the onset of an era of cardiac arrhythmia examination using deep learning technology, have been released.

It is noteworthy that, through the COVID-19 pandemic, the direction of public healthcare and medical services has shifted from face-to-face to contactless services. Moreover, the level of interest in self-healthcare through continuous management has increased in addition to short-term treatments. Furthermore, companies are attempting to implement a paradigm shift using AI and IoT technologies to provide comprehensive healthcare and health promotion services.

The trend in South Korea shows that a greater emphasis is placed on self-healthcare than DTx, which is a central axis in global digital healthcare services. In operation are Health&u^®^ (The Join Inc.) and Biogram^®^ (Healthmax Co., Ltd.), which provide healthcare services through links to wearable devices, and InBody^®^, an app developed by a company with the same name that specializes body composition analyses. Cashwalk^®^, an app used to pay cash for the number of steps counted using a built-in function without the need for a wearable device, is also popular among users. Web portal and internet information-mediating service providers NAVER^®^ and Kakao^®^ recently established digital healthcare research centers, and they are developing a broad range of digital healthcare services focusing on service commercialization [15]. This is because the current focus is placed on preventive care, although the companies are also likely bound by the remote medical service-related laws, systems, and regulations of South Korea in providing services.

As previously mentioned, the Korean Health Promotion Institute (KHPI) developed a public mobile healthcare platform in 2016, and has provided digital healthcare services to adults since then. Currently, digital healthcare services are provided through a link to various wearable devices based on user-friendly apps targeting adult users, children and teenagers, and, now (more recently), senior citizens aged 65 years (or older).

### 2.3. Recent Outcomes of Digital Healthcare Service for Senior Citizens Which Was Implemented by the Korea Health Promotion Institute (KHPI)

In the field of public digital healthcare service, a policy is being actively promoted to expand the phases of the AI–IoT-based healthcare project for senior citizens aged 65 years or older through collaboration with local public health centers in South Korea. Since the end of December 2022, approximately 45,000 senior citizens nationwide have been using the service, justifying the very high level of interest in the service [16]. This is also the only digital healthcare service developed for and provided to a specific target.

In general, public health centers in local communities implement various health promotion projects. While most services are provided through citizens visiting the centers, expansion to group management services has been attempted as the health promotion project was further expanded in the 2010s. With the exception of mobile healthcare services, however, the service is still being provided face-to-face with citizens visiting the centers. As it is necessary to overcome human-, time-, and space-related limitations and achieve the efficient operation of local public health centers in line with the prolongation of the COVID-19 pandemic, AI–IoT-based healthcare services for senior citizens have been developed based on a contactless healthcare service model for public health centers to provide healthcare services continuously to senior citizens in local communities. Specifically, Bluetooth-based smart healthcare devices (automatic blood pressure meters, glucometers, smart bands, scales, screen-type AI speakers) are provided to senior citizens aged 65 years or older who have access to smartphones depending on their health risk factors. In addition, using Today’s Health app^®^, which was developed based user interface (UI) and user experience (UX) technologies for senior citizens only, healthcare missions have been assigned and contactless health consulting services have been provided.

A study on the effectiveness of project participation conducted by Hwang Jeong-hae reported the following positive results [17]. To measure the objective impact of the pilot project, the pre- and post-data from the 24 public health centers that participated in the initial pilot project were utilized and analyzed using a paired *t*-test. The post-evaluation of the subjects included in the analysis revealed that a total of 9566 individuals completed the service and participated for more than 5 months. The analysis demonstrated that the AI–IoT-based senior health care project exhibited a statistically significant improvement in health. Improvements were observed across all areas of healthy living practices, with the highest enhancement seen in the nutrition scores. Physical activity showed the following ranking: walking, moderate intensity, and strength training. From examining the changes in the major clinical values, the average body mass index (BMI) significantly decreased, and this effect was also observed in blood pressure. However, there was a slight increase in blood sugar levels 2 h after a meal. The frailty score, determined by maintaining an overall frailty score, social frailty score, and cognitive function score, exhibited a high improvement rate of 80%, confirming the effectiveness of the service. To understand the service’s performance, and the experiences and perceptions of the pilot project service providers, an online survey targeted 112 project participants from 24 public health centers involved in the initial pilot project. The average number of seniors managed per participant in the project was 148.7, with an average of 261.2 in rural areas, 217.0 in large cities, and 83.4 in small and medium-sized cities, revealing a significant regional disparity. Compared to the subjects in the existing home visit health care project, those discovered in this pilot project showed better transmission of health measurement data, a higher mission implementation rate, and a relatively smaller distribution of frailty groups. In many cases, the number of visits increased or remained unchanged due to non-face-to-face pilot projects, face-to-face evaluation, or device linkage issues. Results from the satisfaction evaluation of the three systems used to carry out the business indicated that each system was helpful in executing the pilot projects and participating in the process. However, if problems arose, the speed of response was relatively limited. The overall satisfaction with the pilot project was high, and according to project quality evaluation standards, the results were ranked as follows: effectiveness–accessibility–appropriateness–efficiency–timelines–responsiveness. While this pilot project utilizing AI-IoT technology can be considered an effective strategy due to its high accessibility, it is relatively weak in terms of the responsiveness to the needs of the target audience, making it challenging to promote the project in the current COVID-19 situation.

Based on a policy study, Park Na-yeong et al. also reported systolic blood pressure, non-fasting glucose level, and body mass index (BMI) improvements in subjects registered in 2022 who had participated in the service for at least 6 months (participation periods of 6, 9, 12, and 18 months), and demonstrated that the effect was maintained over the long term [16]. In addition, the necessity of the project was cited by mentioning the long-term, continuous effect on balance (standing on one leg with the eyes closed). The satisfaction score of the participants was also high (rated at 90.1 points on average).

As it promotes changes in health behaviors through mobile channels, this project is expected to serve as a policy implementation measure complying with the vision of HP2030 to contribute to national health improvements by providing healthcare services to senior citizens and providing customized services centering on local public health centers, thus extending the citizens’ years of healthy life. The AI-IoT-based project started in November 2020 and has entered its third year as of 20 December 2022.

In addition to the short-term effects, the project also examined its medium- to long-term effects. The analysis included a total of 23,207 participants who had been involved for at least 6 months out of the participants from the first to third year. To understand the changes in health outcomes and health behaviors through the AI-IoT-based project, the study looked at changes in health before and after the project (at 6, 9, 12, and 18 months). However, since the number of participants in the post-evaluation varied by time point, an unbalanced panel model was used for the analysis. To examine the net effects of the service, the pre-evaluation and post-evaluation of the participants who participated at the 6-, 9-, 12-, and 18-month time points were compared, as comparing the overall pre-evaluation participants with different post-time point participants directly could lead to selection bias. Therefore, while some participants may not have participated in the post-evaluation at the 6-month time point, they may have been evaluated at the 9-month time point. This assumption was made based on the idea that they were still receiving the service in the long term, even if their measurements were missing at a particular time point. The outcome measures included blood pressure, postprandial glucose level, dizziness, subjective health status, and health behaviors (walking, moderate-intensity physical activity, muscle-strengthening exercises, healthy eating). The average pre-intervention blood pressure of all the subjects was in the prehypertension range (133.1/78.8 mmHg), but it improved to normal blood pressure (127.7/75.6 mmHg) at all the post-intervention evaluations and was maintained up to 18 months. Looking at the distribution of blood pressure according to the evaluation time points, the proportion of subjects with normal blood pressure increased from 45.4% at baseline to 48.3% at 18 months, while the proportion of those with hypertension decreased from 30.2% at baseline to 27.5% at 18 months. Meanwhile, the average postprandial glucose level did not change significantly across all subjects, but it improved significantly (from a mean of 249.9 mg/dL at baseline to 191.3 mg/dL at 6 months) and was maintained up to 18 months in those classified as having diabetes according to the glucose criteria. The frailty scores were measured for overall frailty, social frailty, and cognitive function. The overall frailty score improved from 1.4 to 1.0, the social frailty score improved from 0.9 to 0.5, and the cognitive function improved from 4.2 to 4.5. The participants were further analyzed based on their health status, and the intervention showed positive effects in all the groups. Particularly, the frail group with poor health status showed the most significant improvements, with a 1.7-point improvement in overall frailty, a 1.2-point improvement in social frailty, and a 1.0-point improvement in cognitive function [18]. Despite the positive results listed above, a strategy is still needed for the health-vulnerable class experiencing difficulties participating in digital healthcare, i.e., those without smartphones or subject to limited data use attributed to the possession of low-end smartphone models, or enrollment on lowest-rate plans. A solution was found with the use of an AI speaker. Following the upgrade of the Android-based AI speaker to a display in 2021, Today’s Health^®^, an app for senior citizens, could be mounted on the speaker screen. As a result, senior citizens from the vulnerable class can participate in the project without limitations. Therefore, the project is expected to produce significant outcomes in 2023.

**Figure 1:**
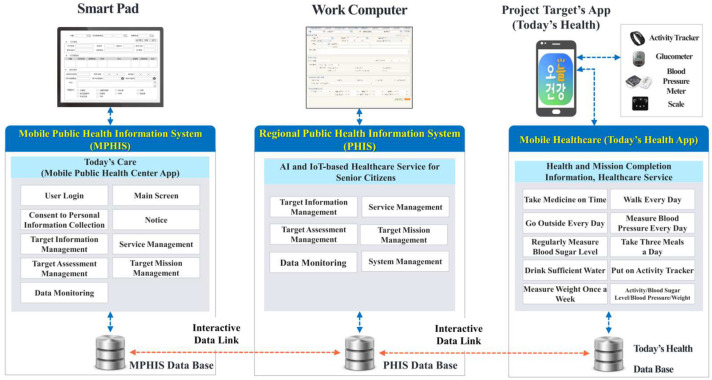
System configuration of artificial intelligence internet of things (AI-IoT)-based healthcare project for senior citizens.

**Figure 2:**
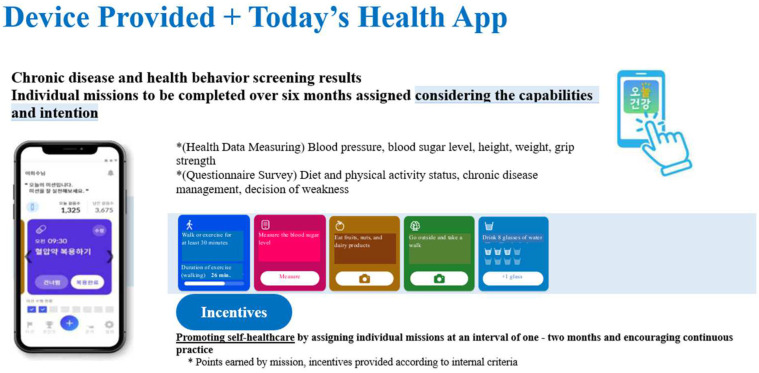
Screen of “Today’s health app” for AI–IoT-based healthcare project for senior citizens.

As mentioned earlier, with the digital healthcare project for senior citizens selected and promoted as a policy task, it is necessary—from the perspective of broadening the service scope—to expand the application of this service to the areas of welfare. In addition, through a link to medical services, the data of Today’s Health app can be used as lifelong data by doctors in hospitals. This in turn will lead to the development of high-quality medical services. In other words, a paradigm shift in healthcare services is essential. It will not only play a considerable role in changing the focus of healthcare from treatment to prevention but also contribute to the broadening of the local safety net by providing efficient healthcare services to senior citizens with lowered access to medical services (Figure 1 and Figure 2).

### 2.4. Mid-to-Long-Term Development Strategies for the Style of South Korean Digital Healthcare Services

Experts forecast that South Korea will enter a super-aging society by 2025. So far, the healthcare services (health counseling and education) for senior citizens in local communities have been provided by nurses by visiting their homes. Nowadays, the provision of smart healthcare services and the use of AI technology must be expanded to reduce the medical expenses of senior citizens. As high-quality service provision is possible only when supported by technological development, it is necessary to configure a network environment without limitations in terms of smartphone specifications and data use. The positive effect of a digital healthcare service on senior citizens aged 65 years or older was reported previously. However, there are still some people in a health-vulnerable class experiencing difficulties participating in the digital healthcare project, i.e., those without smartphones or subject to limited data use because their smartphones are low-end models or on the lowest rate plans. It is necessary to continue developing new strategies that will ameliorate issues associated with these people.

**Figure 3:**
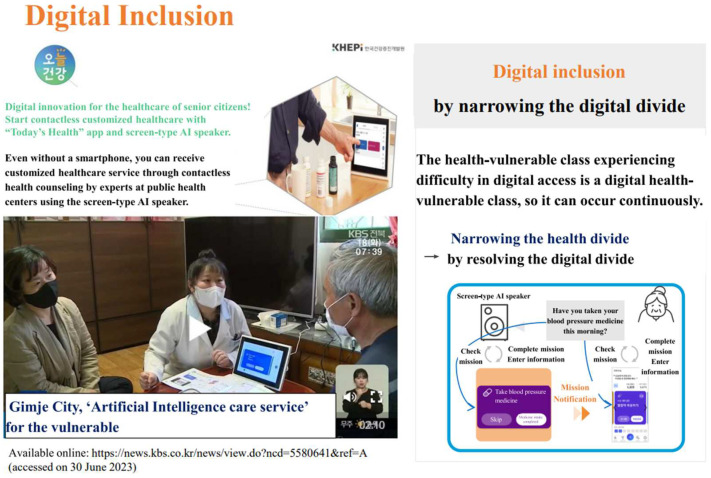
Digital inclusion. Available online: https://news.kbs.co.kr/news/view.do?ncd=5580641&ref=A (accessed on 30 June 2023).

To this end, the screen-type AI speaker must be continuously upgraded and developed into a user-friendly assistance device equipped with various health contents. In other words, by providing interactive service rather than one-sided information provision, the link to and service use by senior citizens will be increased. Likewise, Today’s Health app^®^ community will help senior citizens recognize information more conveniently and swiftly. In addition, the screen-type AI speaker can provide emergency and safety management services. In addition to its use for healthcare purposes, it can serve as a basis for achieving “aging in place” through a link to a wider range of care services including those in the welfare field. Furthermore, if the health measurements and data of Today’s Health app^®^, i.e., individual lifelog data, are used in connection to medical services, such as My Data PHR, this will enable the provision of high-quality medical services (Figure 3). In fact, South Korea has actively pursued non-face-to-face healthcare management services since 2016. In 2020, in response to COVID-19, the country implemented AI- and IoT-based projects targeting the elderly. However, elderly individuals with relatively low digital literacy find it challenging to utilize mobile apps and devices. Therefore, it is necessary to develop policies and programs that not only focus on digital education but also establish support systems by connecting service providers or social support networks to encourage participation. Additionally, it is important to develop technologies that are user-friendly for the elderly, allowing them to control the technology comfortably and primarily adopt familiar technologies based on their previous experiences. This approach will facilitate the acceptance and long-term usage of such services [19].

From another perspective, a new healthcare service using senior towns can be considered. An increasing number of senior citizens who have financial capacity are considering spending their twilight years in senior towns. However, the use of private residential facilities for senior citizens in Korea has not been popularized as these are premium-level facilities and the entry barrier is high. Therefore, as the government needs to focus on the health-vulnerable class, the onset of the era of healthcare services focusing on senior towns is approaching.

By recognizing and preparing for the trends of the current times and the paradigm shift in the healthcare field, an efficient public service can be provided for the health-vulnerable class. Moreover, through cooperation with the private sector, the government will be able to offer a broad range of health content services.

## 3. Conclusions

This study discussed the field application of the AI- and IoT-based healthcare project for senior citizens (which started in the second half of 2020) and related study results and proposed plans for the diffusion of the project.

The Fourth Industrial Revolution, which has been accelerated as a result of the global COVID-19 pandemic, introduced numerous changes to our society at large. In addition, internet-based digital technology has revolutionized our lives. It increases the efficiency, transparency, and convenience of a range of tasks ranging from interactions through communication to financial services. Likewise, a data-based public health and medical revolution is currently occurring.

Therefore, in the public sector, effort must be expended to prepare digital healthcare strategies including the user-oriented UI and UX upgrade for Today’s Health App^®^ and improve the efficiency and convenience of health-measuring devices for the health-vulnerable class. At the same time, while strengthening the capabilities of senior citizens for self-healthcare and health promotion, it must strive to provide emotional support using the screen-type AI speaker and, configure an environment to contribute to the improvement of senior citizens’ quality of life. Based on the results and effects of the project promoted so far, the public sector must develop and improve public healthcare service policies through cooperation with private companies.

In addition, there is a need to integrate AI- and IoT-based elderly healthcare management services with the existing offline visit-based healthcare service model. Furthermore, active consideration should be given to collaboration with the welfare sector in order to provide integrated digital-based integrated care services. In a study by Park Nayeong (2023), it was highlighted that in order for the elderly to adopt and participate in digital new technologies in the long term, an integrated operating system should be established. This is because a mixed service approach that appropriately mediates face-to-face and non-face-to-face services has the greatest impact in terms of effectiveness evaluation, as the service recipients are similar [20]. In the local community, a mixed service approach combining face-to-face and non-face-to-face services can increase the participation and utilization rates of public health management services and, furthermore, provide high-quality services by collaborating with the welfare sector. In this context, it is important to consider the elderly’s smartphone specifications, data usage constraints, and network environment to ensure that high-quality healthcare management services are available to the public. Going forward, digital-based healthcare management services are expected to expand further. However, if the economic aspects are not supported by appropriate regulations, health disparities may widen. Therefore, it is important to consider a digital health policy approach from the perspective of health equity.

## Data Availability

Data sharing not applicable.
